# Biofilm inhibition and bactericidal activity of NiTi alloy coated with graphene oxide/silver nanoparticles via electrophoretic deposition

**DOI:** 10.1038/s41598-021-92340-7

**Published:** 2021-07-07

**Authors:** Sirapat Pipattanachat, Jiaqian Qin, Dinesh Rokaya, Panida Thanyasrisung, Viritpon Srimaneepong

**Affiliations:** 1 Department of Prosthodontics, Faculty of Dentistry, Chulalongkorn University, Bangkok, Thailand; 2 Metallurgy and Materials Science Research Institute (MMRI), Chulalongkorn University, Bangkok, Thailand; 3 International College of Dentistry, Walailak University, Bangkok, Thailand; 4 Department of Microbiology and Research Unit on Oral Microbiology and Immunology, Faculty of Dentistry, Chulalongkorn University, Bangkok, Thailand

**Keywords:** Health care, Materials science

## Abstract

Biofilm formation on medical devices can induce complications. Graphene oxide/silver nanoparticles (GO/AgNPs) coated nickel-titanium (NiTi) alloy has been successfully produced. Therefore, the aim of this study was to determine the anti-bacterial and anti-biofilm effects of a GO/AgNPs coated NiTi alloy prepared by Electrophoretic deposition (EPD). GO/AgNPs were coated on NiTi alloy using various coating times. The surface characteristics of the coated NiTi alloy substrates were investigated and its anti-biofilm and anti-bacterial effect on *Streptococcus mutans* biofilm were determined by measuring the biofilm mass and the number of viable cells using a crystal violet assay and colony counting assay, respectively. The results showed that although the surface roughness increased in a coating time-dependent manner, there was no positive correlation between the surface roughness and the total biofilm mass. However, increased GO/AgNPs deposition produced by the increased coating time significantly reduced the number of viable bacteria in the biofilm (*p* < 0.05). Therefore, the GO/AgNPs on NiTi alloy have an antibacterial effect on the *S. mutans* biofilm. However, the increased surface roughness does not influence total biofilm mass formation (*p* = 0.993). Modifying the NiTi alloy surface using GO/AgNPs can be a promising coating to reduce the consequences of biofilm formation.

## Introduction

Currently, nickel-titanium (NiTi) alloy is used in many biomedical devices due to its mechanical properties and biocompatibility. Because of its versatility, NiTi alloy has been used for biomedical devices, such as vascular stents, staples, catheter guide wires, bone fixtures, including orthodontic wires, and endodontic instruments. However, biofilm formation on medical/dental devices is a major reason for their clinical complications or failure. Among dental treatments, fixed orthodontic appliances are commonly recognized as a source for potential high caries risk because they have many inaccessible sites and eventually develop biofilm-forming areas^1–3^.

Graphene is a carbon nanomaterial that has a single layer of *sp*^2^- hybridized carbon atoms condensed in a hexagonal honey-comb appearance with two-dimensional structures. Graphene oxide (GO) is a highly oxidized form of graphene composed of a graphene sheet and its oxygenated portion. Many GO oxidized functional groups can be used to create many compound materials possessing exceptional improvements in mechanical, physical, and biological characteristics, especially when incorporating GO into many nanocomposites^4–6^. The previous studies have shown that coating NiTi alloy with a GO nanocomposite using electrophoretic deposition lowered the coefficient of friction and increased corrosion resistance and surface hardness^7,8^. In addition to improved mechanical properties, the antibacterial effects of GO were increased by adding nanoparticles, such as silver nanoparticles (AgNPs), gold nanoparticles (AuNPs), or copper oxide nanoparticles (CuONPs), on the GO functionalized surface^9,10^. In industry, graphene-metal nanocomposites have been used in various manners, including using graphene nanosheets with gold or silver nanoparticles as heat transfer technology^11,12^. In addition to their biocompatibility, metal-based (either Au or Ag) graphene has been investigated for biomedical use due to their unique thermal, mechanical, and optical properties, such as a photothermal therapy (PTT) in cancer treatment or a biosensor as a diagnostic device^13^. Although the GO nanoparticles have excellent biocompatibility, these materials can be toxic in certain conditions. Long-term graphene exposure can cause inflammation and cell death, depending on the concentration, duration, and treatment used^14^. Among many useful nanoparticles, silver nanoparticles (AgNPs) are a promising material for medical use because of their exceptional antibacterial effect along with good biocompatibility to human cells^15,16^. Moreover, the antibacterial action of AgNPs can be increased when combined with other materials, especially graphene oxide, and this combination yields a better antibacterial effect compared with pure AgNPs^17^.

Based on the ecological plaque hypothesis, plaque accumulation under some conditions such as sugar consumption, decreases the biofilm pH which leads to an ecological shift in resident microbiota to cariogenic bacteria^18,19^. *Streptococcus mutans* (*S. mutans*) is a major cariogenic bacteria due to its three main virulence characteristics, acid-tolerance, acid-production, and biofilm formation^19^. Among the different surface modification methods, Electrophoretic deposition (EPD) is one of the most useful and predictable coating approaches because it results in many outstanding characteristics, i.e. good surface coverage, controllable deposited film thickness, dense packing, and one-step processing^20^. Although the previous studies found that a longer EPD coating time produces better mechanical surface properties, it also increased surface roughness^7,8,21^ which could affect the formation of a biofilm on the surface of a NiTi alloy. Therefore, the aim of this study was to determine the influence of a GO/AgNPs coated NiTi alloy with different EPD coating durations on biofilm formation, including its bactericidal effect. The null hypotheses of this study were that the GO/AgNPs coating would have no bactericidal effect on the biofilm and the increased coating surface roughness would not influence biofilm mass formation.

## Results

### Surface characterizations

#### Raman spectra, surface morphology and elemental analysis

**Figure 1:**
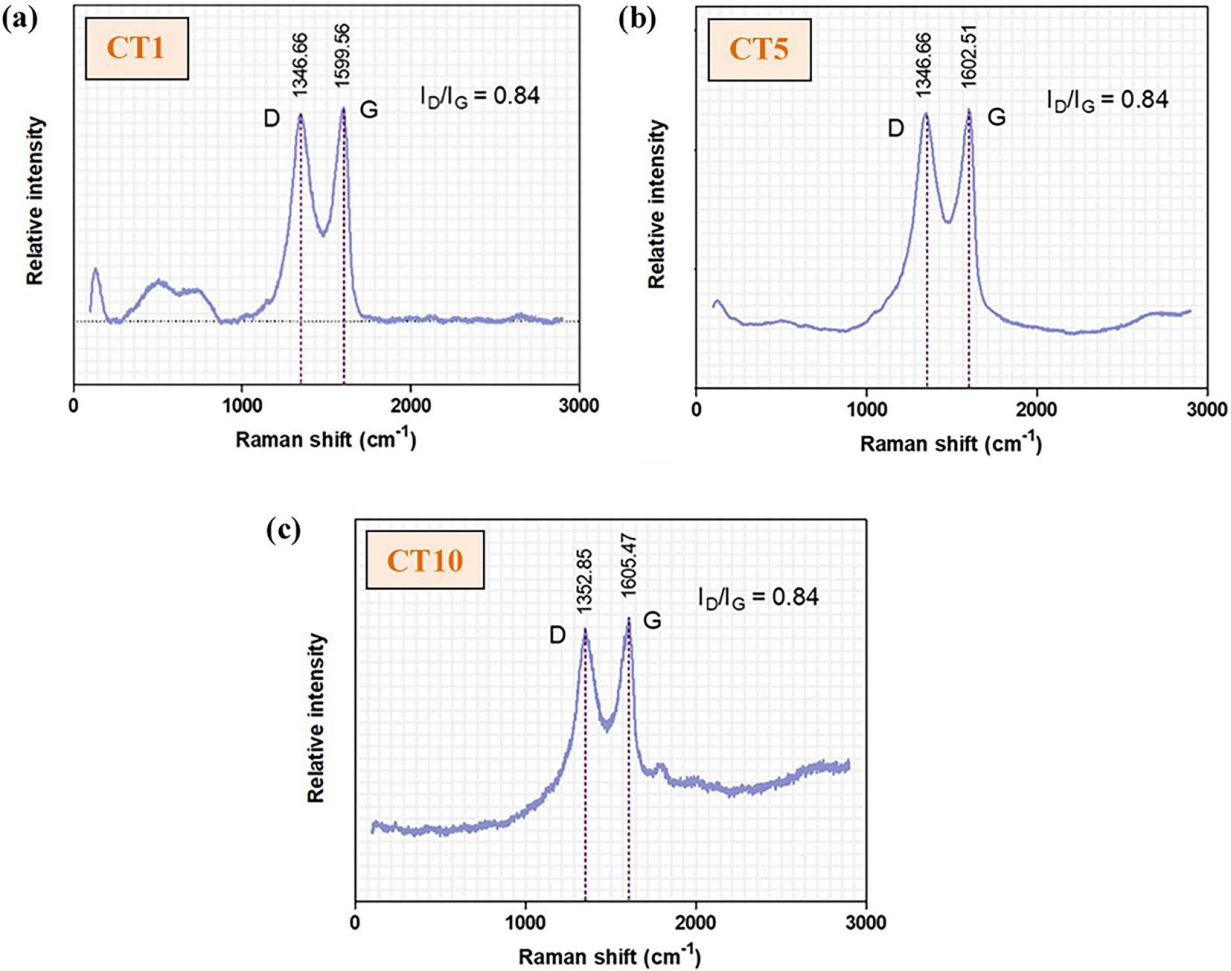
Raman spectroscopy of the GO/AgNPs coated NiTi alloy samples coated using 3 coating times: (**a**) CT1 = 1 min, (**b**) CT5 = 5 min, and (**c**) CT10 = 10 min.

**Figure 2:**
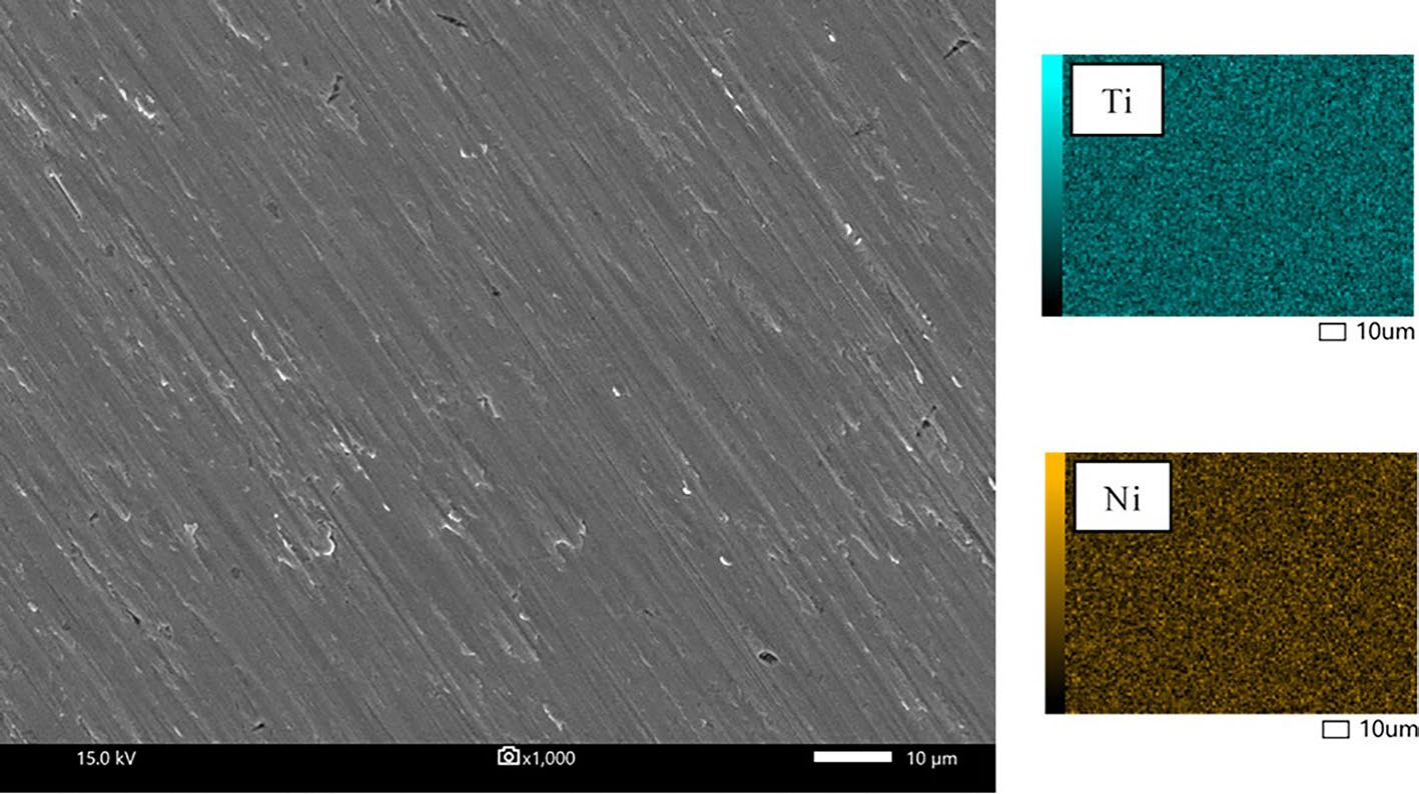
SEM images and EDS mapping of the uncoated NiTi alloy sample.

**Figure 3:**
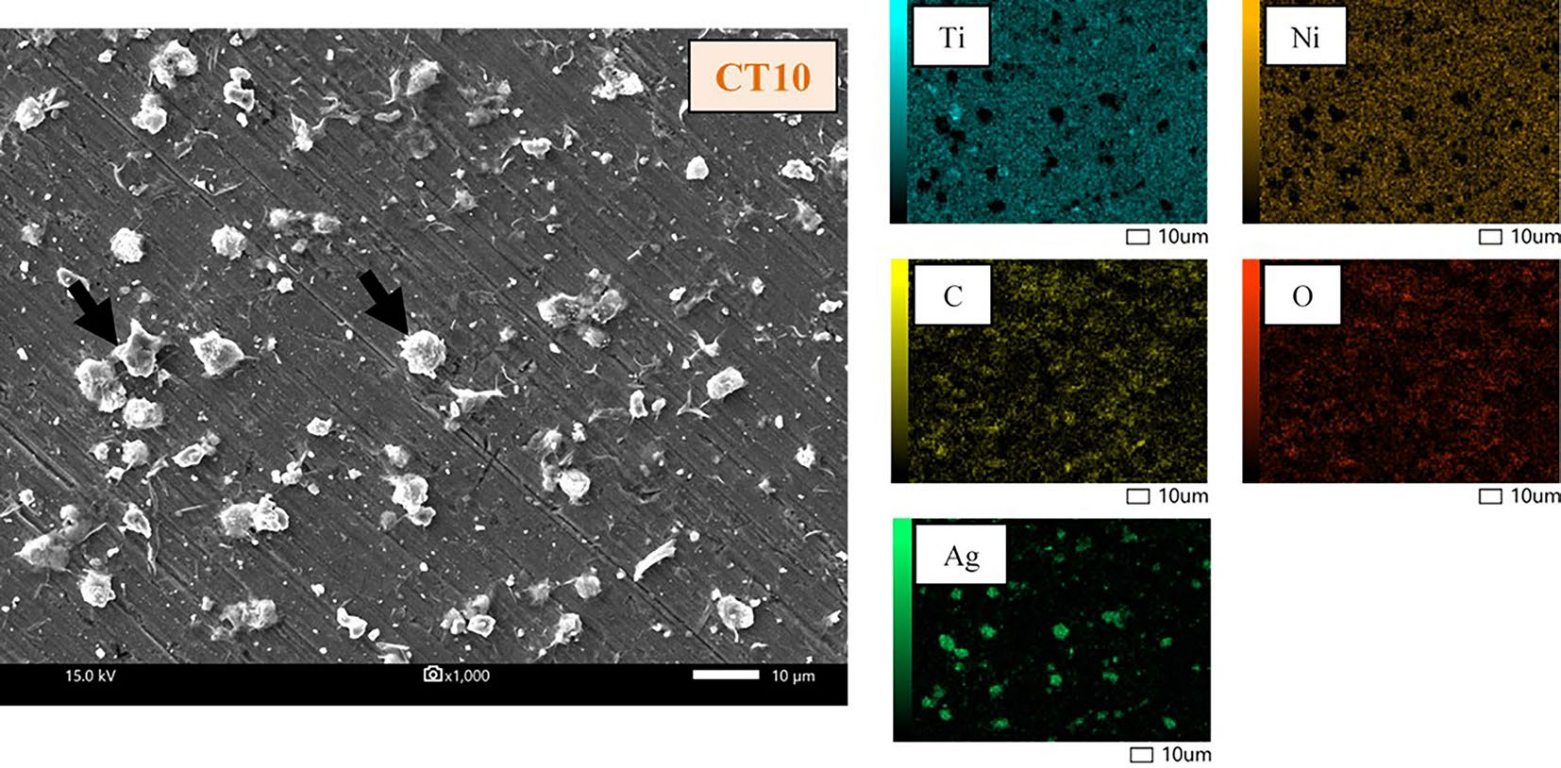
SEM images and EDS mapping of the 10 min GO/AgNPs coated NiTi alloy sample (CT10). Black arrows indicate the GO assembly with the AgNPs.

The characteristics of the GO deposited on the surface of the NiTi alloy were identified and confirmed by the existence of D and G bands on the Raman spectra (Fig. 1). The intensity ratios of the D bands and G bands (I_D_/I_G_) of all coating groups were 0.84. Note that not all the SEM images and EDS mappings of specimen groups are presented in this study, except for the uncoated (Fig. 2) and 10-min (CT10) samples (Fig. 3). The coated surfaces of each experimental group were evaluated at randomly selected areas using EDS (Table 1). The results indicated that the amount of Ti and Ni detected on the coated surface decreased with increased coating time.

**Table 1:** Surface elemental analysis by energy disperse spectroscopy (EDS).

Samples	Elements (wt%)				
	Titanium	Nickel	Carbon	Oxygen	Silver
Uncoated	50.65	49.35	–	–	–
CT1 (1 min)	42.89	50.50	2.60	3.01	1.01
CT5 (5 min)	38.72	45.76	5.51	7.16	2.85
CT10 (10 min)	34.41	38.56	8.33	11.08	7.62

#### Surface roughness (Ra)

**Figure 4:**
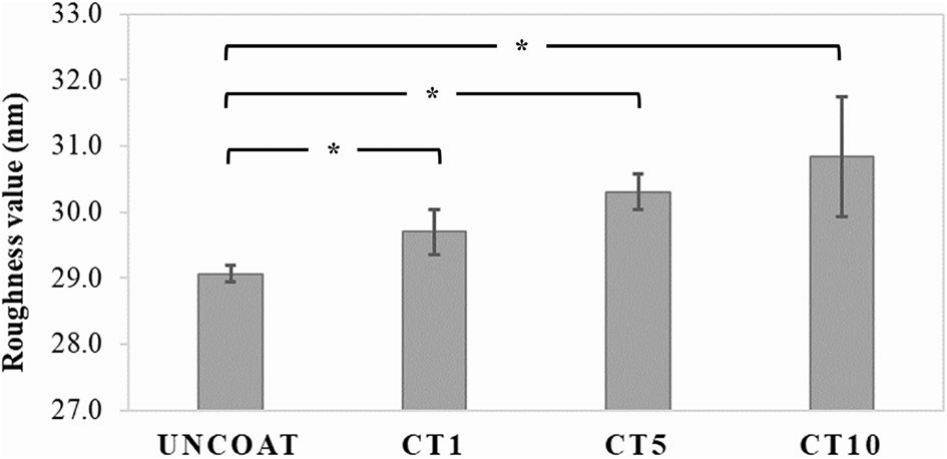
Surface roughness (Ra, nm) of the uncoated NiTi alloy sample and the GO/AgNPs coated NiTi alloy samples with different coating times. Asterisk (*) represents a significant difference (*p* < 0.05) by Welch’s ANOVA and Game-Howell multiple comparison tests.

The mean values and standard deviation (nm) of surface roughness are presented in Fig. 4. The surface of each coating group was significantly rougher than that of the uncoated group (*p* < 0.05). Welch’s ANOVA and Game-Howell multiple comparison tests determined that the surface roughness of GO/AgNPs coating was significantly higher compared with the uncoated group (29.061 ± 0.126 nm) followed by the CT1 group (29.697 ± 0.340 nm), CT5 group (30.302 ± 0.267 nm), and the CT10 group (30.833 ± 0.904 nm).

### Biofilm inhibition assay

**Figure 5:**
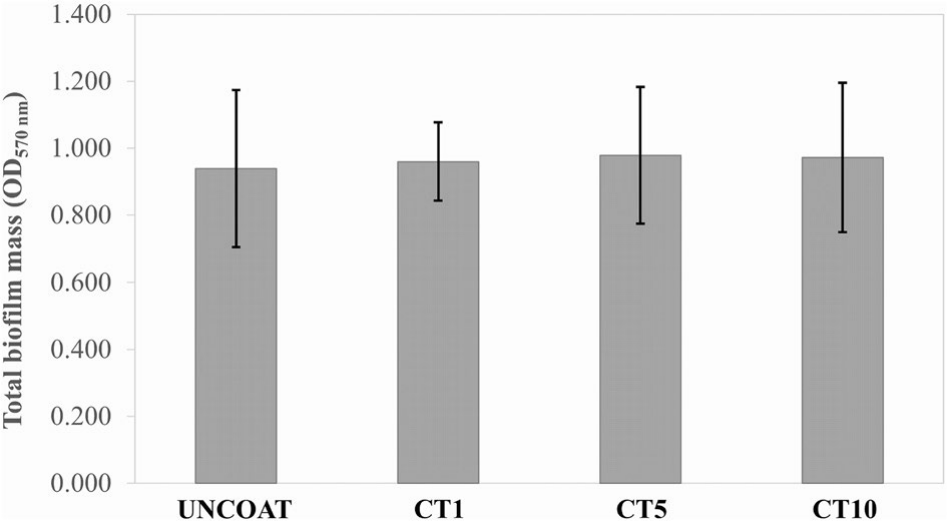
The total biofilm mass (represented as an optical density value at 570 nm) on the surface of the uncoated NiTi alloy sample and the GO/AgNPs coated NiTi alloy samples using different coating times.

A crystal violet (CV) assay was performed to determine the biofilm inhibition effect of the GO/AgNPs coated NiTi alloy produced in different durations. Figure 5 presents the mean and standard deviation of the biofilm mass formed on surface of each material (n = 5/group). The results revealed that there was no significant difference in the total biofilm mass between the groups (*p* = 0.993), however, the surface roughness tended to increase coating time-dependently.

### Examination of the bactericidal effect on S. mutans biofilm

**Figure 6:**
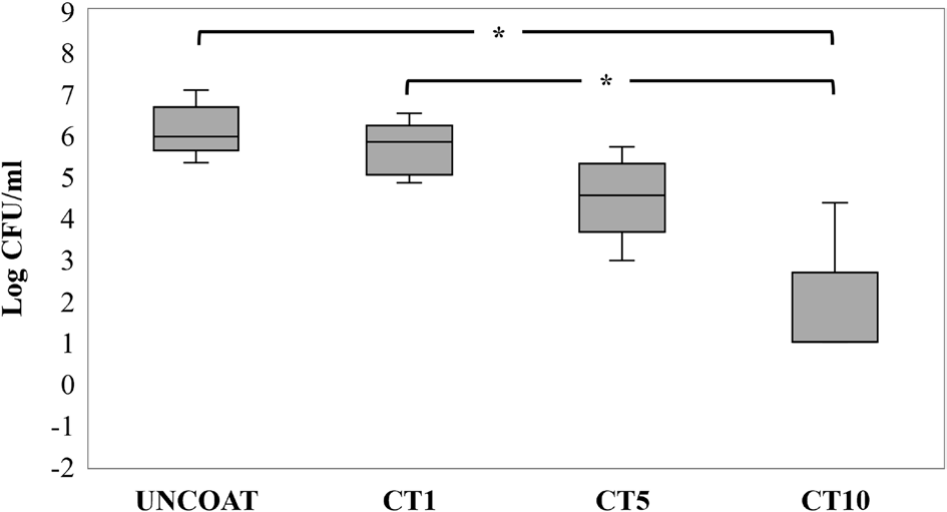
The number of viable bacteria (represented as Log CFU/mL) on the surface of the uncoated NiTi alloy sample and the GO/AgNPs coated NiTi alloy samples using different coating times. Asterisk (*) represents a significant difference (*p* < 0.05) by Kruskal–Wallis H test and Dunn’s test.

A colony counting assay was performed to determine the number of viable cells in the biofilm adhered on the surface of the tested samples. The mean and standard deviation of the log CFU/mL in each group (n = 5/group) are found in Fig. 6. The number of viable cells in the CT10 group (0.871 log CFU/mL) were significantly lower than those of the uncoated (6.108 log CFU/mL) and CT1 groups (5.674 log CFU/mL) (*p* < 0.05), however, there was no significant difference in the number of viable cells between the CT1, CT5, (4.493 log CFU/mL), and uncoated groups (*p* = 0.798).

**Figure 7:**
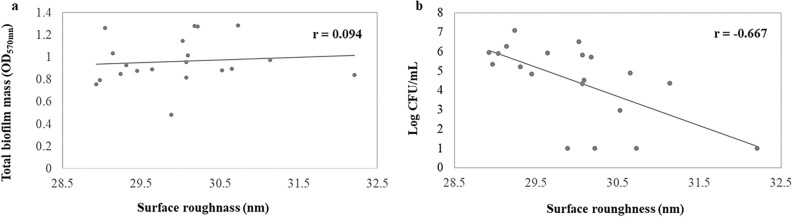
Statistical correlation between (**a**) The surface roughness and the total biofilm mass and (**b**) The surface roughness and log CFU/mL. Each data point represents the material surface roughness value (x-axis) and the biofilm mass amount or CFU (y-axis) on the coated NiTi alloy specimen.

Correlation analysis was performed to determine the relationship between the surface roughness and the biofilm mass. This analysis indicated that there was no correlation between the surface roughness and the biofilm mass formation (*r* = 0.094, *p* = 0.692). In contrast, the correlation analysis between the surface roughness and log CFU/mL of viable cells demonstrated a negative correlation (*r* =  − 0.667, *p* = 0.001) (Fig. 7).

## Discussion

A dental biofilm commonly forms on hard surfaces, e.g., tooth surfaces, dental prosthesis, and restorative materials and exposes the teeth and periodontal tissues to a greater amount of bacteria metabolites that cause dental diseases, such as dental caries, gingivitis, or periodontitis. Biofilm formation occurs by a complex mechanism that involves many surrounding factors, including surface morphology, hydrophobicity, and temperature^22,23^. Different environment surface conditions also directly affect the biofilm nature and development. The material surfaces including surface topography and surface roughness have an important effect on bacterial adhesion and subsequent biofilm formation in all degrees down to the micrometric or nanometric scale^24^.

The previous studies successfully produced GO/AgNPs coated NiTi alloys with improved coating surface mechanical and physical properties^7,8,21^. These studies demonstrated that a longer coating time results in better material properties and tends to increase surface roughness, which may affect biofilm formation. Using EPD, electrochemical deposition immobilizes and deposits many metal nanoparticles from a liquid medium to a conductive-solid substrate to create nanoscale surface roughness. Moreover, the thickness and density of the deposited metal particles on substrates increased with the deposition duration. We found that the roughness of coated NiTi alloy substrates ranged from 29.697 to 30.833 nm, which was higher than the roughness of the uncoated NiTi samples.

Although some studies demonstrated that a surface roughness of material involved in microbial adhesion and biofilm formation^25–27^, this correlation is unresolved and is not a linear relationship^24,28^. This study showed that there was no difference in biofilm mass formation on the NiTi alloy surface between the GO/AgNPs coated NiTi groups and the uncoated NiTi group. Moreover, the correlation analysis found no relationship between the surface roughness and biofilm mass formation. Although the surface roughness on biofilm formation influences biofilm accumulation, the hydrophobicity of a GO coated surface has a greater effect on biofilm accumulation^29,30^. Not only does GO increase the hydrophobicity, but also AgNPs increase surface hydrophobicity^31^. Therefore, the hydrophobicity of the GO/AgNPs has a greater influence on biofilm formation compared with surface roughness. The previous studies have proposed that a rougher surface is more easily adhered to by bacteria compared with a smooth surface because it has numerous extended surfaces for bacterial adhesion and biofilm formation. Rougher surfaces protect bacteria from shear forces and promote their attachment, leading to mature biofilm formation^25,26,32^. However, this concept has been questioned because it cannot be applied to nanoscale surface roughness. Furthermore, other studies found that a rougher surface had a higher attraction to bacteria compared with a smoother surface only in the initial stages, approximately 2–6 h of incubation, and after that surface roughness did not affect the number of adherent bacteria^33,34^.

The finding that there was no difference in the total biofilm mass corresponds with those of previous in vivo and in vitro studies^33–36^. These studies reported that there was no significant difference in adherent biofilm mass between 4 h and 5 d of incubation, and the increased surface nanoscale roughness of many dental materials, such as zirconia, acrylic resin, or titanium, did not affect the number of colonized bacteria. A possible reason is the proceeding maturation of the biofilm. The influence of a material’s nanoscale rough surface would be compensated for by biofilm maturation, especially in the early stage called the “conditioning film or pellicle formation”. When the biofilm initially develops, it begins with the adsorption of organic and inorganic substances to a solid surface. When the surfaces and microorganisms are exposed to an aqueous surrounding that contains a high level of the organic material such as tears, blood, or saliva, they will be covered with adsorbed organic particles within seconds. Many proteins are adsorbed to form a conditioning film on a surface known as the acellular pellicle or acquired pellicle^37^. The presence of this pellicle can mask the rough substratum and makes it smoother compared with the underlying coarse area^22,33–36^. The proceeding maturation of oral biofilm and the antibacterial properties of GO/AgNPs may also affect the total biofilm mass. The bactericidal effect results in dead bacteria that gradually smoothens the rough surface of the corrugated GO. Farid et al. reported that these dead cell layers may also act as a conditioning layer that can mask the GO nanosheet edges that prevents the direct contact between other live cells and GO^38^. These could explain why we found no significant change in total biofilm mass formation between the experimental groups, despite that the surface roughness tended to increase in a coating time-dependent manner. This finding will accept the null hypothesis that the increased coating surface roughness would not influence biofilm mass formation.

We found that NiTi alloy coated with GO/AgNPs for 10 min had the highest bactericidal action on the biofilm (almost completely removed viable *S. mutans* compared with the uncoated surface). Therefore, these results indicate that this GO/AgNP coating has an excellent antibacterial effect. The results demonstrated that the number of viable *S. mutans* decreased in the biofilm formed on the surface of GO/AgNPs coated NiTi alloys with increased coating times. This reduction could be due to the higher amount of GO and AgNPs deposited on the NiTi substrates, which corresponds to previous studies suggesting that the antibacterial effect of GO and AgNPs increases in concentration- or contact time-dependent manners^26,39^. A higher number of GO functional groups and nano-sharp edges can kill bacteria via mechanical and chemical direct contact^40^ together with the high amounts of released Ag^+^ from AgNPs^17^ that enhances the bactericidal effect. These results indicated that the surface roughness of the GO/AgNPs coated NiTi specimens generated using EPD did not induce more biofilm mass formation compared with the uncoated NiTi alloy. In contrast, the higher amount of GO/AgNPs resulted in a higher bactericidal effect on the biofilms. Although GO/AgNPs on NiTi are biocompatible to human pulp fibroblasts and studies have reported good biocompatibility of GO^8,13^, other studies have found that GO/Au or GO/Ag led to cell impairment and graphene-based materials in particular generated harmful reactive oxygen species (ROS)^41,42^. Therefore, the null hypothesis that GO/AgNPs would have no bactericidal effect on biofilm is rejected.

Although AgNPs are a well-known anti-bacterial material, silver ions may be toxic. AgNPs toxicity is directly related to the amount of free Ag^+^ ions released into medium. Ag^+^ ions can cause soft tissue discoloration (Argyria), contact dermatitis, or genotoxicity or neurotoxicity^43^. The long-term biocompatibility of GO/AgNPs needs to be further investigated. Because the biocompatibility of GO/AgNPs is unresolved, other possible nanocomposites should be considered. Many kinds of Ag-based nanocomposites have been engineered instead of using GO/Ag for antimicrobial activity, such as Ag-ZnO and Ag-TiO_2_^44,45^. Moreover, studies have produced biosynthesized nanoparticles for biomedical uses, such as FeO and Cr_2_O_3_ nanoparticles with improved biocompatibility^46–48^. These nanoparticles should be considered as future alternatives. Additionally, this study only investigated the antibacterial effects on *S. mutans* mono-species biofilm, which does not replicate all aspects of the oral environment*.* Therefore, further investigations on multispecies biofilms and clinical studies are required before GO/AgNPs surface coating could be safely used long term clinically.

## Methods

### Sample and graphene oxide/silver nanoparticle solution preparation

Sixty 6 × 6 × 1 mm medical grade NiTi alloy (55.7 wt% Ni, 44.8 wt% Ti, and 0.2 wt% other elements, Baoji Seabird Metal Material Co., Ltd., China) plates were prepared. The samples were polished with silicon carbide paper (400–2000 grit) and ultrasonically cleaned by sequentially immersing them in acetone, alcohol, and deionized (DI) water solutions for 10 min. Finally, the samples were etched using Kroll’s reagent composed of 2 ml 40% nitric acid, 4 ml 40% hydrofluoric acid and 994 ml deionized (DI) water and ultrasonicated in DI water for 10 min.

GO powder (Nanjing Jing Ji Cang Nano Technology Co., Nanjing, China) (average diameter of 50 µm) was used in this study. The GO was produced by graphite oxidation using concentrated sulfuric acid and potassium permanganate. One-hundred ml GO aqueous solution (0.05 mg/ml) was prepared in DI water and ultrasonicated for 3 h and a 100 ml silver nitrate (AgNO_3_) solution (0.05 mg/ml) was prepared and ultrasonicated for 20 min. The GO and AgNO_3_ solutions were mixed and ultrasonicated for 30 min. The AgNPs solution was produced by chemical reduction using trisodium citrate (Na_3_C_6_H_5_O_7_) as the stabilizing agent^49^. The GO/AgNO_3_ solution was heated at 80 °C for 1 h and 10 ml 0.05 mg/ml Na_3_C_6_H_5_O_7_ was added dropwise. The solution was kept at 80 °C for 1 h and ultrasonicated for 20 min at room temperature to obtain a homogenous GO/AgNO_3_ mixture.

### Electrophoretic deposition (EPD) of graphene oxide/ silver nanoparticles

The NiTi alloy sample surfaces were coated with the GO/AgNPs solution using EPD. The anodic deposition was performed on the NiTi samples at the anode (+) with a constant voltage of 20 V for 1, 5, and 10 min referred as the CT1, CT5, and CT10 groups, respectively (n = 5/group^7^). Platinum was used as the cathode (−). The coated NiTi alloy samples were rinsed with DI water and dried at room temperature for 24 h and at 80 °C for 5 h. The surface characteristics and antimicrobial and biofilm inhibition effects of the coated samples were evaluated.

### Surface characterizations

#### Surface analysis, surface morphology, and elemental analysis

The surface coating Raman spectra were obtained using Raman spectroscopy (LabRAM HR Evolution, Horiba Scientific Inc., New York, USA) at room temperature with a grating of 1200 gr/mm, 200-µm slit, and 532 nm laser wavelength. The surface morphology observations and elemental analysis of the uncoated and coated NiTi alloy samples were repeated using a Scanning Electron Microscope (SEM) (JSM-IT300, JEOL Ltd., Japan) at 30 kV and Energy Dispersive X-ray Spectrometer (EDS) (Oxford X-Max 20, Oxford, UK), respectively.

#### Surface roughness measurement

The surface roughness was investigated using a surface-contact profilometer device (Taylor Scan 150, Taylor Hobson Ltd., Leicester, UK). The samples were traced 11 times mesiodistally at the center of the samples in the same direction and 1 mm in length (Gaussian filter at 0.08, spacing at 1 µm, and a speed of 1500 µm/s) before and after coating. The mean surface roughness (Ra) was calculated from 10 measured areas between the 11 traced lines for each specimen.

### Bacteria and growth condition

*Streptococcus mutans* UA159 was used in this study. *S. mutans* frozen stock was thawed and inoculated in brain heart infusion (BHI, HiMedia, India) agar and incubated at 37 °C with 5% CO_2_ for 24 h. A single colony was inoculated into BHI broth and incubated at 37 °C with 5% CO_2_ overnight. The optical density of the overnight culture was measured by a spectrophotometer (Thermo Scientific GENESYS 20, USA) at a 600 nm wavelength (OD_600nm_) and then adjusted to an OD_600nm_ of 0.1. The adjusted-culture was incubated at 37 °C with 5% CO_2_ for 2 h until reaching the log-phase (approximately 10^7^ colony-forming unit/milliliter (CFU/mL)) that was used for the biofilm formation assay.

### Biofilm formation

There were 4 biofilm formation groups (n = 5) including (1) uncoated group, (2) 1 min-coated group (CT1), (3) 5 min-coated group (CT5), and (4) 10 min-coated group (CT10). Each coated sample was mounted into a 6-well microtiter plate well (Sigma-Aldrich, USA) and sterilized using low-temperature hydrogen peroxide plasma (Johnson & Johnson, Thailand). The log-phase bacteria were re-suspended in BHI broth with 5% sucrose (final concentration ≈ 10^7^ CFU/mL)^29^. Ten ml bacterial suspension was added into each well to cover the mounted sample. The plate was incubated at 37 °C with 5% CO_2_ for 24 h to allow mature biofilm formation.

### Biofilm inhibition evaluation

Biofilm inhibition was investigated using a crystal violet (CV) assay as previously described^29,50^. This method quantifies the total biofilm mass based on the CV dye’s binding ability to the extracellular polymeric substances (EPS) and both live and dead bacterial cells^51^. The 24 h biofilms were gently washed with phosphate-buffered saline (PBS, pH = 7.4) to remove unbound bacteria. The biofilms were fixed with 70% ethanol at room temperature for 20 min. Subsequently, the 70% ethanol was removed and 200 µl 1% (w/v) crystal violet solution (Sigma-Aldrich, USA) was added into each well. The biofilms were stained for 20 min and washed 3 times with PBS to remove the excess dye. Subsequently, 500 µl 30% acetic acid was added to each well and incubated for 10 min to elute the stain. After incubation, 200 µl acetic acid was transferred to a new 48-well plate. The absorbance of the de-staining solution was measured at 570 nm using a microplate reader (BioTek Epoch2 microplate reader, USA). The optical density of the de-staining solution represented the biofilm mass. The experiments were independently repeated 5 times.

### Examination of the bactericidal effect on the S. mutans biofilm

A colony counting assay^29^ was performed to quantify the number of viable bacteria in the biofilm*.* The 24 h biofilms were washed with PBS to remove unbound bacteria. The biofilms were detached from the samples by sonicating them for 10 min in PBS and then tenfold serially diluted. One-hundred µl from each dilution was spread on BHI agar and incubated at 37 °C with 5% CO_2_ for 24 h. The bacterial colonies were counted and displayed as log CFU/mL. The experiments were independently repeated 5 times.

### Statistical analysis

The Shapiro–Wilk and Levene’s tests were performed to determine the data normality and homogeneity of variances, respectively, using SPSS (statistical analysis software version 22.0, Chicago, IL, USA). One-way ANOVA and Tukey’s post-hoc analysis was used to analyze the surface roughness and the biofilm mass, and the Kruskal–Wallis H test and Dunn’s test were used to analyze the number of viable cells. Correlation analysis was performed to determine the relationships between surface roughness, biofilm mass, and number of viable bacteria. The level of significance was set at *p* < 0.05.

### Ethical approval

This article does not contain any studies with human participants or animals performed by any of the authors.

## Conclusions

Within the limitations of our controlled in vitro experiment on mono-species biofilm, the increased surface roughness of GO/AgNPs coated NiTi alloys did not increase the amount of total biofilm mass formed on the surface of NiTi alloy. In contrast, higher amounts of GO/AgNPs on the coated NiTi alloys generated an increased anti-bacterial effect. Therefore, GO/AgNPs can be a promising anti-bacterial surface coating material on NiTi alloy for dental uses.

## References

[1] Richter A, Arruda A, Peters M, Sohn W (2011). Incidence of caries lesions for patients treated with comprehensive orthodontics. Am. J. Orthod. Dentofacial. Orthop..

[2] Tufekci E, Dixon JS, Gunsolley JC, Lindauer SJ (2011). Prevalence of white spot lesions during orthodontic treatment with fixed appliances. Angle Orthod..

[3] Shukla C, Maurya R, Singh V, Tijare M (2016). Evaluation of changes in Streptococcus mutans colonies in microflora of the Indian population with fixed orthodontics appliances. J. Dent. Res..

[4] Papageorgiou DG, Kinloch IA, Young RJ (2017). Mechanical properties of graphene and graphene-based nanocomposites. Prog. Mater. Sci..

[5] Novoselov KS (2012). A roadmap for graphene. Nature.

[6] Kuilla T (2010). Recent advances in graphene based polymer composites. Prog. Polym. Sci..

[7] Rokaya D, Srimaneepong V, Qin J, Siralertmukul K, Siriwongrungson V (2019). Graphene oxide/silver nanoparticle coating produced by electrophoretic deposition improved the mechanical and tribological properties of NiTi alloy for biomedical applications. J. Nanosci. Nanotechnol..

[8] Srimaneepong V, Rokaya D, Thunyakitpisal P, Qin J, Saengkiettiyut K (2020). Corrosion resistance of graphene oxide/silver coatings on Ni–Ti alloy and expression of IL-6 and IL-8 in human oral fibroblasts. Sci. Rep..

[9] Wang L, Hu C, Shao L (2017). The antimicrobial activity of nanoparticles: present situation and prospects for the future. Int. J. Nanomed..

[10] Kumar P, Huo P, Zhang R, Liu B (2019). Antibacterial properties of graphene-based nanomaterials. J. Nanomater..

[11] Mbambo MC (2020). Remarkable thermal conductivity enhancement in Ag-decorated graphene nanocomposites based nanofluid by laser liquid solid interaction in ethylene glycol. Sci. Rep..

[12] Mbambo MC (2020). Thermal conductivity enhancement in gold decorated graphene nanosheets in ethylene glycol based nanofluid. Sci. Rep..

[13] Darabdhara G (2019). Ag and Au nanoparticles/reduced graphene oxide composite materials: synthesis and application in diagnostics and therapeutics. Adv. Colloid Interface Sci..

[14] Bostan HB (2016). Cardiotoxicity of nano-particles. Life Sci..

[15] Rizzello L, Pompa PP (2014). Nanosilver-based antibacterial drugs and devices: mechanisms, methodological drawbacks, and guidelines. Chem. Soc. Rev..

[16] Noronha VT (2017). Silver nanoparticles in dentistry. Dent. Mater..

[17] Jaworski S (2018). Graphene oxide-based nanocomposites decorated with silver nanoparticles as an antibacterial agent. Nanoscale Res. Lett..

[18] Pitts NB (2017). Dental caries. Nat. Rev. Dis. Primers.

[19] Marsh PD (2006). Dental plaque as a biofilm and a microbial community: implications for health and disease. BMC Oral Health.

[20] Besra L, Liu M (2007). A review on fundamentals and applications of electrophoretic deposition (EPD). Prog. Mater. Sci..

[21] Rokaya D, Srimaneepong V, Qin J, Thunyakitpisal P, Siralertmukul K (2019). Surface adhesion properties and cytotoxicity of graphene oxide coatings and graphene oxide/silver nanocomposite coatings on biomedical NiTi alloy. Sci. Adv. Mater..

[22] Cheng Y, Feng G, Moraru CI (2019). Micro- and nanotopography sensitive bacterial attachment mechanisms: a review. Front Microbiol..

[23] Garcia-Gonzalo D, Pagán R (2015). Influence of environmental factors on bacterial biofilm formation in the food industry: a review. Postdoc. J..

[24] Desrousseaux C, Sautou V, Descamps S, Traoré O (2013). Modification of the surfaces of medical devices to prevent microbial adhesion and biofilm formation. J. Hosp. Infect..

[25] Quirynen, M., Dierickx, K. & van Steenberghe, D. in *Handbook of Bacterial Adhesion: Principles, Methods, and Applications* (eds Yuehuei H. An & Richard J. Friedman) 91–102 (Humana Press, 2000).

[26] Yadav N (2017). Graphene oxide-coated surface: inhibition of bacterial biofilm formation due to specific surface-interface interactions. ACS Omega.

[27] Zaugg LK (2017). Determinants of biofilm formation and cleanability of titanium surfaces. Clin. Oral Implants Res..

[28] Ferreira Ribeiro C (2016). Initial oral biofilm formation on titanium implants with different surface treatments: an in vivo study. Arch. Oral Biol..

[29] Agarwalla SV (2019). Hydrophobicity of graphene as a driving force for inhibiting biofilm formation of pathogenic bacteria and fungi. Dent. Mater..

[30] De-la-Pinta I (2019). Effect of biomaterials hydrophobicity and roughness on biofilm development. J. Mater. Sci. Mater. Med..

[31] Wang J, Yi G (2019). Flexible and superhydrophobic silver nanoparticles decorated aligned silver nanowires films as surface-enhanced raman scattering substrates. Nanoscale Res. Lett..

[32] Teughels W, Assche N, Sliepen I, Quirynen M (2006). Effect of material characteristics and/surface topography on biofilm development. Clin. Oral Implants Res..

[33] Morgan TD, Wilson M (2001). The effects of surface roughness and type of denture acrylic on biofilm formation by Streptococcus oralis in a constant depth film fermentor. J. Appl. Microbiol..

[34] Nascimento C (2013). Oral biofilm formation on the titanium and zirconia substrates. Microsc. Res. Techn..

[35] Al-Ahmad A (2010). Biofilm formation and composition on different implant materials in vivo. J. Biomed. Mater. Res. B.

[36] Yu P (2016). Influence of surface properties on adhesion forces and attachment of streptococcus mutans to zirconia in vitro. BioMed. Res. Int..

[37] Baker JL, Bor B, Agnello M, Shi W, He X (2017). Ecology of the oral microbiome: beyond bacteria. Trends Microbiol..

[38] Farid MU, Guo J, An AK (2018). Bacterial inactivation and in situ monitoring of biofilm development on graphene oxide membrane using optical coherence tomography. J. Memb. Sci..

[39] Jin J, Zhang L, Shi M, Zhang Y, Wang Q (2017). Ti-GO-Ag nanocomposite: the effect of content level on the antimicrobial activity and cytotoxicity. Int. J. Nanomed..

[40] Henriques PC, Borges I, Pinto AM, Magalhães FD, Gonçalves IC (2018). Fabrication and antimicrobial performance of surfaces integrating graphene-based materials. Carbon.

[41] Zhang Y (2010). Cytotoxicity effects of graphene and single-wall carbon nanotubes in neural phaeochromocytoma-derived PC12 cells. ACS Nano.

[42] Zhou X (2014). A quantitative study of the intracellular concentration of graphene/noble metal nanoparticle composites and their cytotoxicity. Nanoscale.

[43] Hadrup N, Sharma AK, Loeschner K (2018). Toxicity of silver ions, metallic silver, and silver nanoparticle materials after in vivo dermal and mucosal surface exposure: a review. Regul. Toxicol. Pharmacol..

[44] Hameed S (2019). Greener synthesis of ZnO and Ag-ZnO nanoparticles using Silybum marianum for diverse biomedical applications. Nanomedicine (Lond).

[45] Jiang X, Lv B, Wang Y, Shen Q, Wang X (2017). Bactericidal mechanisms and effector targets of TiO2 and &nbsp;Ag-TiO2 against Staphylococcus aureus. J. Med. Microbiol..

[46] Aisida SO (2020). Biogenic synthesis of iron oxide nanorods using Moringa oleifera leaf extract for antibacterial applications. Appl. Nanosci..

[47] Sone BT, Manikandan E, Gurib-Fakim A, Maaza M (2016). Single-phase α-Cr2O3 nanoparticles’ green synthesis using Callistemon viminalis’ red flower extract. Green Chem. Lett. Rev..

[48] Thomas B (2019). Antioxidant and photocatalytic activity of aqueous leaf extract mediated green synthesis of silver nanoparticles using passiflora edulis f. flavicarpa. J. Nanosci. Nanotechnol..

[49] Lee PC, Meisel D (1982). Adsorption and surface-enhanced Raman of dyes on silver and gold sols. Am. J. Phys. Chem..

[50] Merritt JH, Kadouri DE, O'Toole GA (2006). Growing and analyzing static biofilms. Curr. Protoc. Microbiol..

[51] Allkja J (2019). Minimum information guideline for spectrophotometric and fluorometric methods to assess biofilm formation in microplates. Biofilm.

